# Supplementary material for: Zfp260 choreographs the early stage osteo-lineage commitment of skeletal stem cells
Source: Nat Commun. 2024 Nov 24;15:10186. doi: 10.1038/s41467-024-54640-0 (PMC11586402; doi:10.1038/s41467-024-54640-0)
Supplement: Supplementary file 1 — Supplementary Information [file 41467_2024_54640_MOESM1_ESM.pdf]

Zfp260 choreographs the early stage osteo-lineage commitment of skeletal stem cells

Supplementary Fig. 1 Cluster annotations and time course analysis of bulk RNA-seq (corresponding to Figure 1)

Supplementary Fig. 2 Subcluster annotations of fracture dataset and trajectory analysis by Monocle 2 (corresponding to Figure 1)

Supplementary Fig. 3 Subcluster annotations of MSFL dataset and trajectory analysis by Monocle 2 (corresponding to Figure 1)

Supplementary Fig. 4 Workflow and gating strategies for FACS (corresponding to Figs. 2, 4, and 7)

Supplementary Fig. 5 Peaks enrichment of Zfp260 detected in the promoter regions of several well-known TFs (corresponding to Figure 3)

Supplementary Fig. 6 Suggested docking for each Zfp260 mutant (corresponding to Figure 6)

Supplementary Fig. 7 AAV9-Ctsk infection efficiency and low-magnification histology (corresponding to Figure 7)

Supplementary Fig. 8 The construction strategy for *Zfp260-flox* mouse (C57BL/6J background)

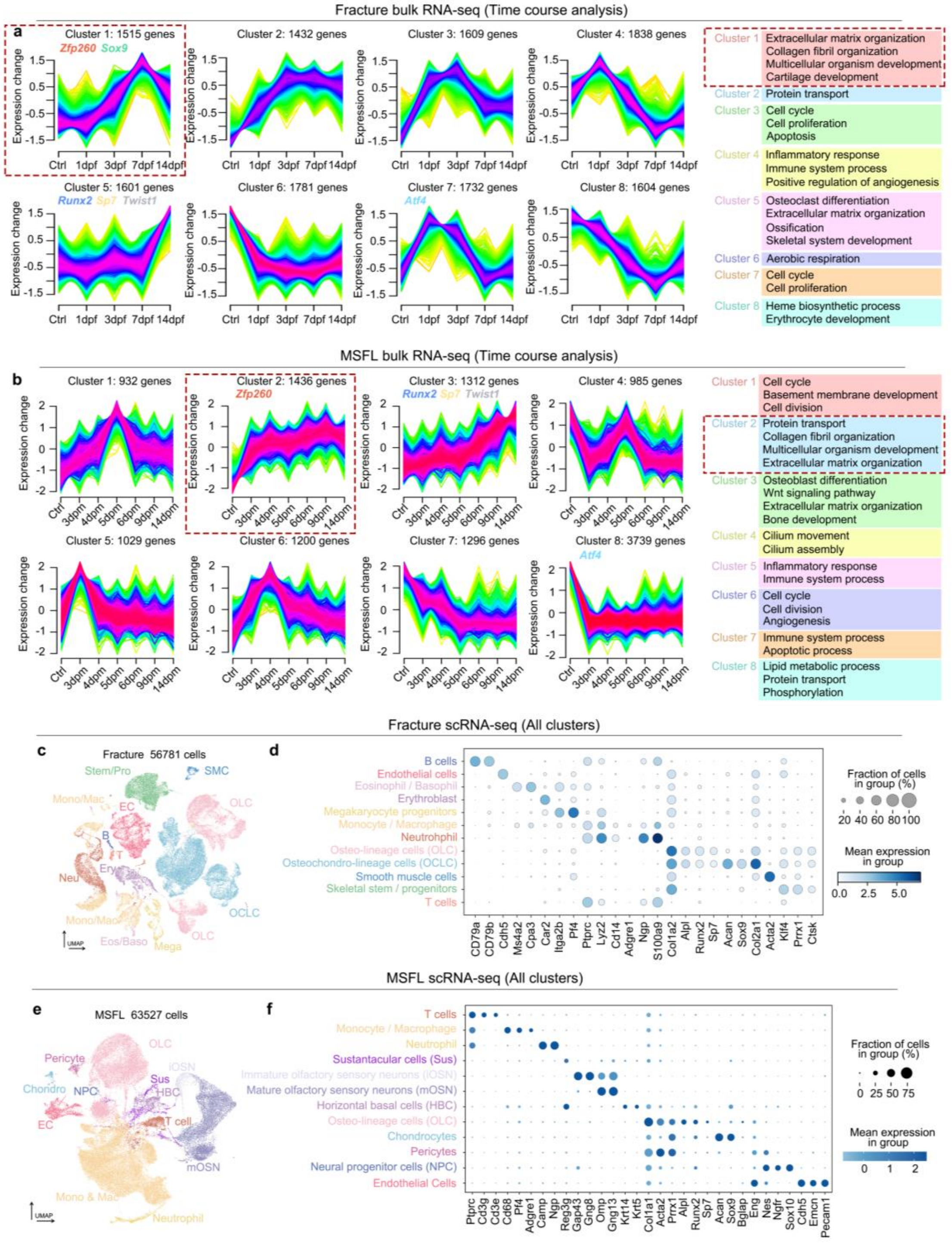

Supplementary Fig. 1 Cluster annotations and time course analysis of bulk RNA-seq (corresponding to Figure 1)

a Time course analysis of fracture dataset GSE152677 (left). The red dotted box indicates the target cluster for the following analysis. The top enriched GO-biological processes of each cluster are displayed (right).

b Time course analysis of MSFL dataset CRA007231 (left). The red dotted box indicated the target cluster for the following analysis. The top enriched GO-biological processes of each cluster are displayed (right).

c Distribution of 56,781 cells from the combination of dataset GSE154247, CD31^{+} and

CD45$^+$ cells (lymphoid and endothelial cells) and Lin$^-$CD140α$^+$ cells (stromal cells) from the mid-shaft of the femurs. In total, 14 clusters were visualized by UMAP. Stem/Pro, stem or progenitor cells; SMC, smooth muscle cells; Mono/Mac, monocytes or macrophages; EC, endothelial cells; OLC, osteo-lineage cells; OCLC, osteochondro-lineage cells; B, B cells; T, T cells; Ery, erythrocytes; Neu, neutrophils; Eos/Baso, eosinophils or basophils; Mega, megakaryocytes.

d Expression patterns of signature genes from **a**.

e Distribution of 63,527 cells from mNM (Ctrl) and newly-formed tissue from postoperative days 3, 6, 9, and 14 of dataset CRA007231. In total, 12 clusters were visualized by UMAP. EC, endothelial cell; iOSN, immature olfactory sensory neuron; mOSN, mature olfactory sensory neuron; NPC, neural progenitor cells; Sus, sustentacular cells.

f Expression patterns of signature genes from **c**.

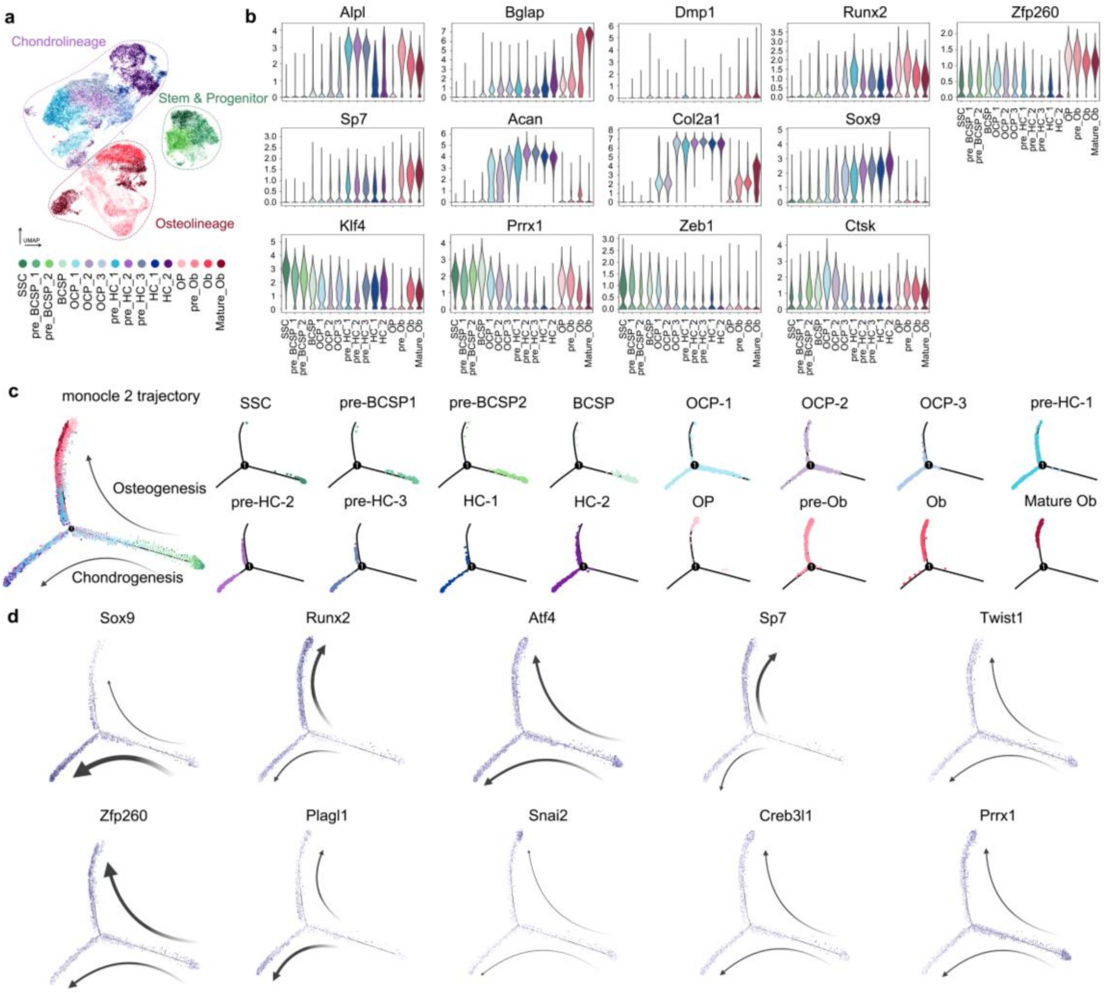

Supplementary Fig. 2 Subcluster annotations of fracture dataset and trajectory analysis by Monocle 2 (corresponding to Figure 1)

a Distribution of 37,053 re-clustered cells from the combination of Stem/Pro, OCLC, and OLC. In total, 14 subclusters are visualized by UMAP projection.

b Violin plots of osteogenic-related, chondrogenic-related, and stemness-related genes in each subcluster.

c Pseudotime lineage trajectory analysis by Monocle 2 demonstrating the relationships of subclusters.

d The expression levels of classical TFs and candidate TFs along the differentiation trajectories, color-coded by their expression levels. The thickness of the arrows depicts the weights of each TF involved in the osteogenic or chondrogenic differentiation process. The start of the arrows indicates the initiation of the TF expression.

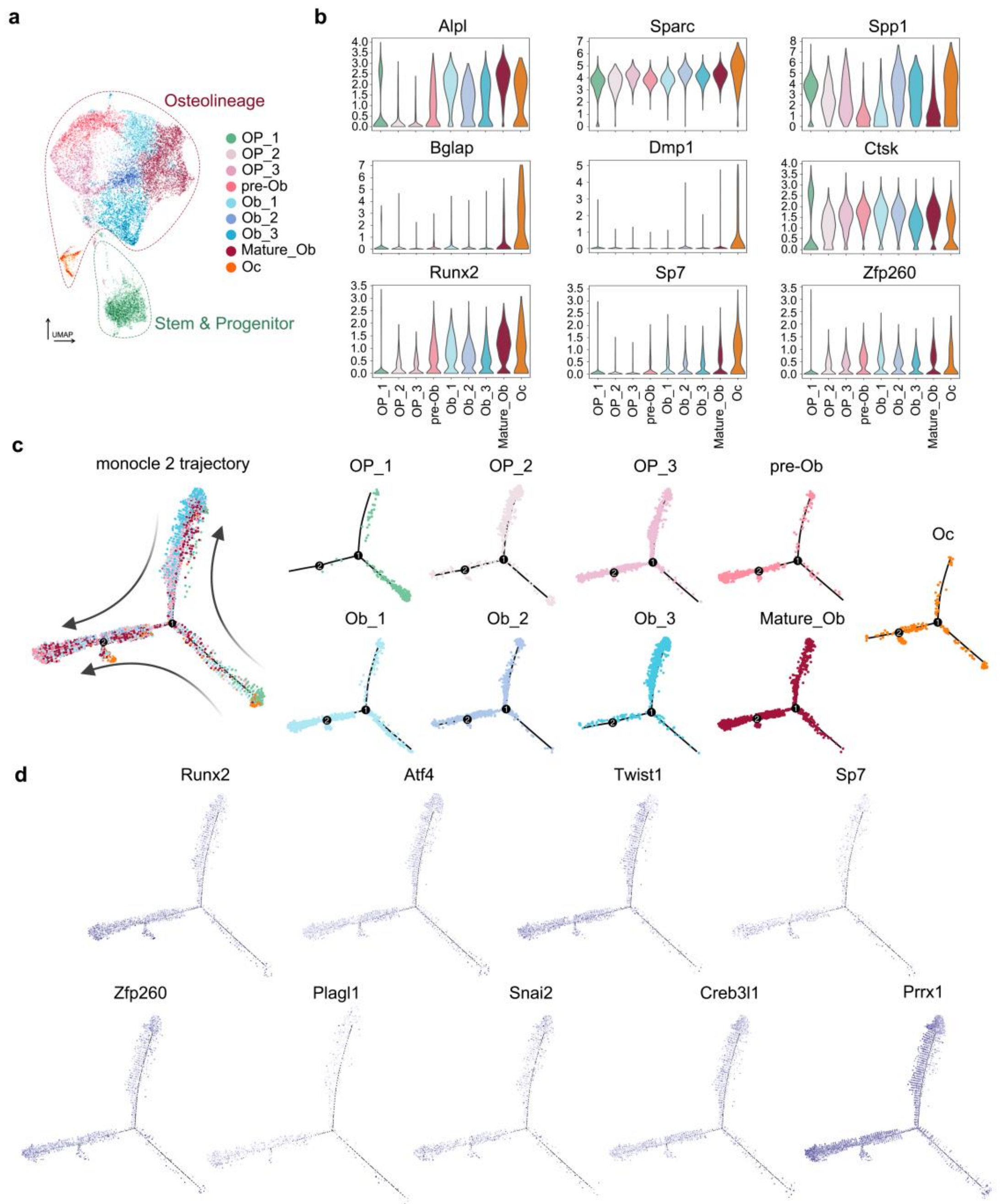

Supplementary Fig. 3 Subcluster annotations of MSFL dataset and trajectory analysis by Monocle 2 (corresponding to Figure 1)

a Distribution of 19,732 re-clustered cells from the OLC. In total, 9 subclusters are visualized by UMAP projection.

b Violin plots of osteogenic-related genes in each subcluster.

c Pseudotime lineage trajectory analysis by Monocle 2 demonstrating the relationships of subclusters.

d The expression levels of classical TFs and candidate TFs along the differentiation trajectories, color-coded by their expression levels.

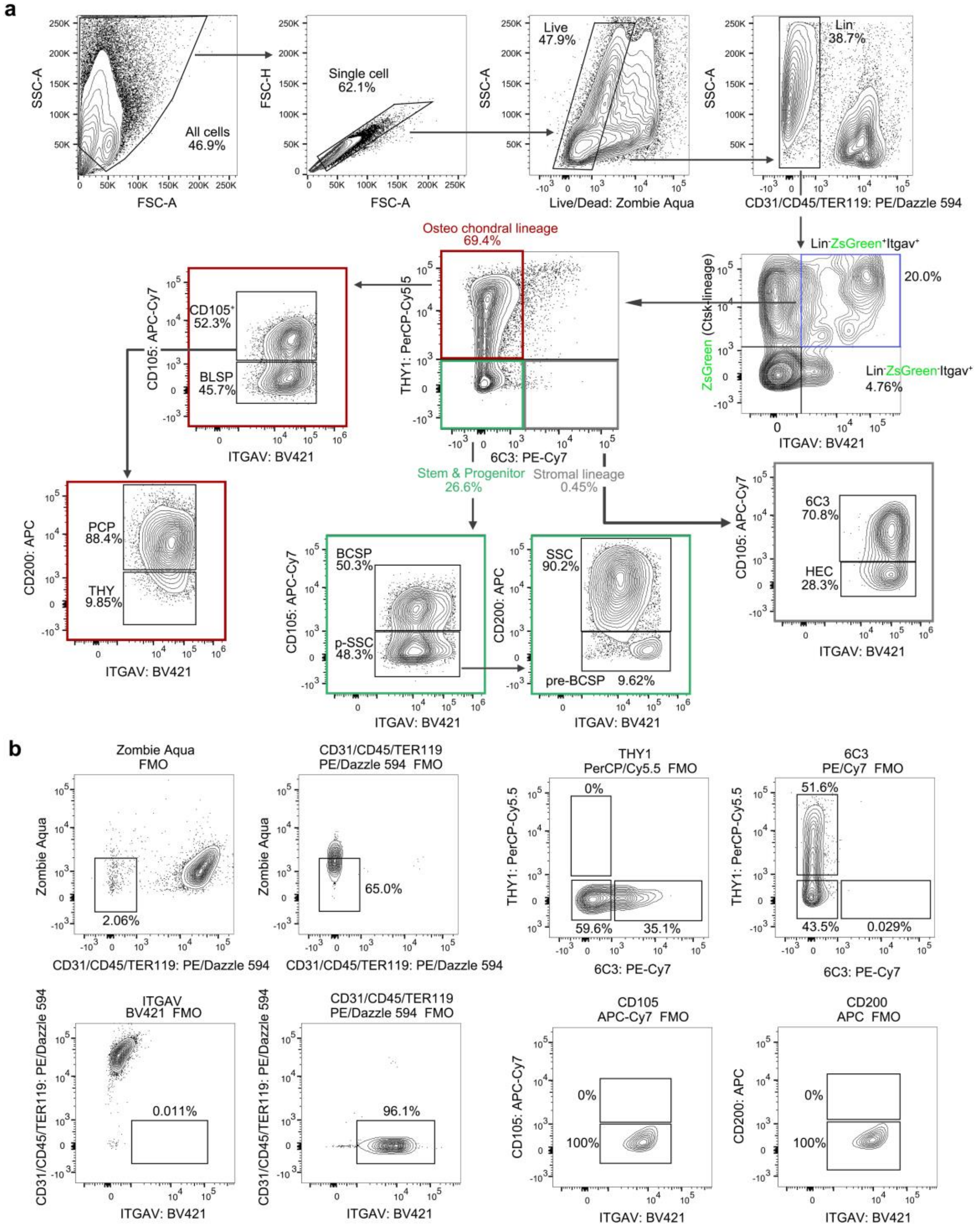

Supplementary Fig. 4 Workflow and gating strategies for FACS (corresponding to Figs. 2, 4, and 7)

a Workflow of FACS to distinguish the subclusters within the SSC hierarchy.

b The positive gates are determined by FMO control.

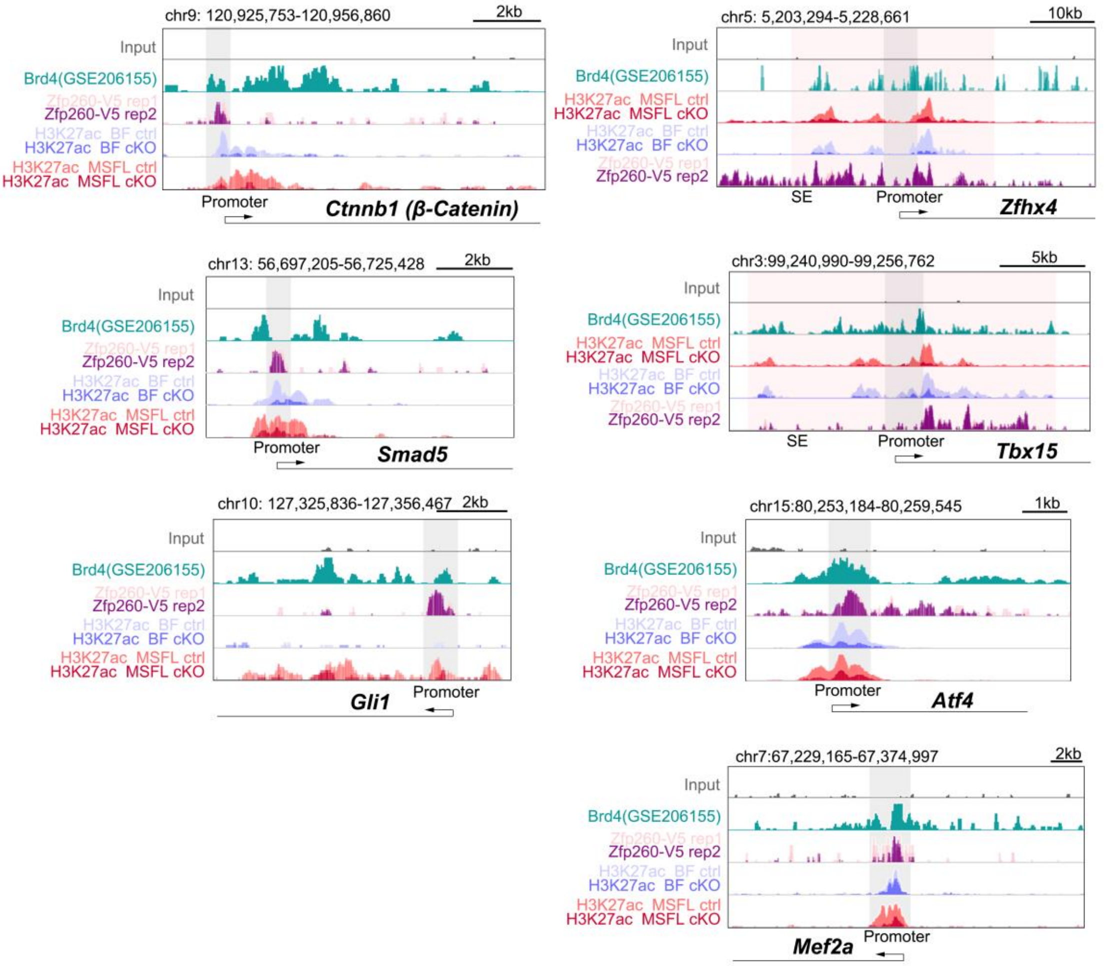

Supplementary Fig. 5 Peaks enrichment of Zfp260 detected in the promoter regions of several well-known TFs (corresponding to Figure 3)

Genome browser view of peak enrichment for H3K4me1, Brd4, H3K27ac, and Zfp260-V5 in the promoter regions of *Ctnnb1*, *Smad5*, *Gli1*, *Atf4*, *Mef2a*, and the super-enhancer regions of *Zfhx4* and *Tbx1*.

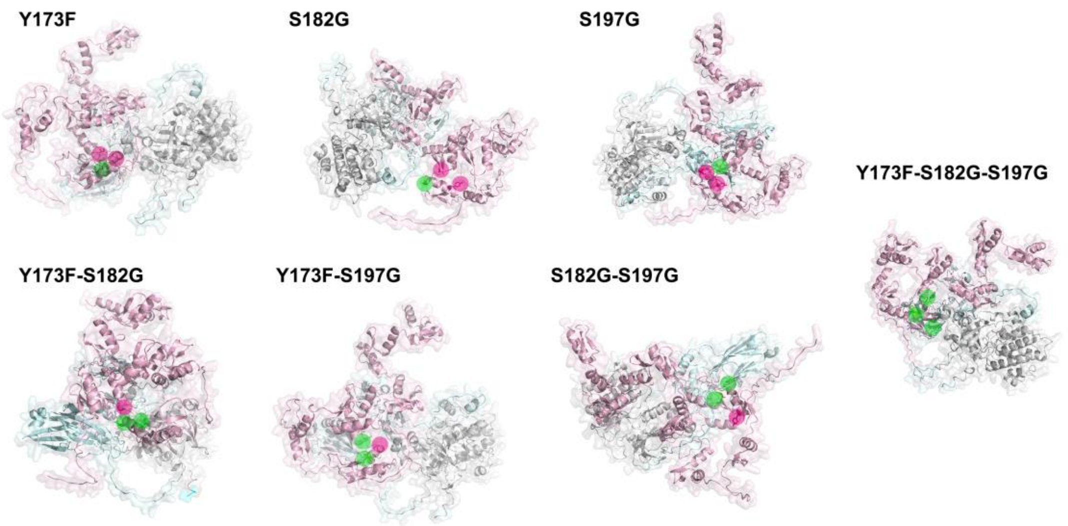

Supplementary Fig. 6 Suggested docking for each Zfp260 mutant (corresponding to Figure 6)

Suggested binding modes of Zfp260-Y173F, Zfp260-S182G, Zfp260-S197G, Zfp260-Y173F-S182G, Zfp260-Y173F-S197G, Zfp260-S182G-S197G, and Zfp260-Y173F-S182G-S197G with Prkca. The magenta and green dots indicate the normal and mutant residues of Zfp260, respectively.

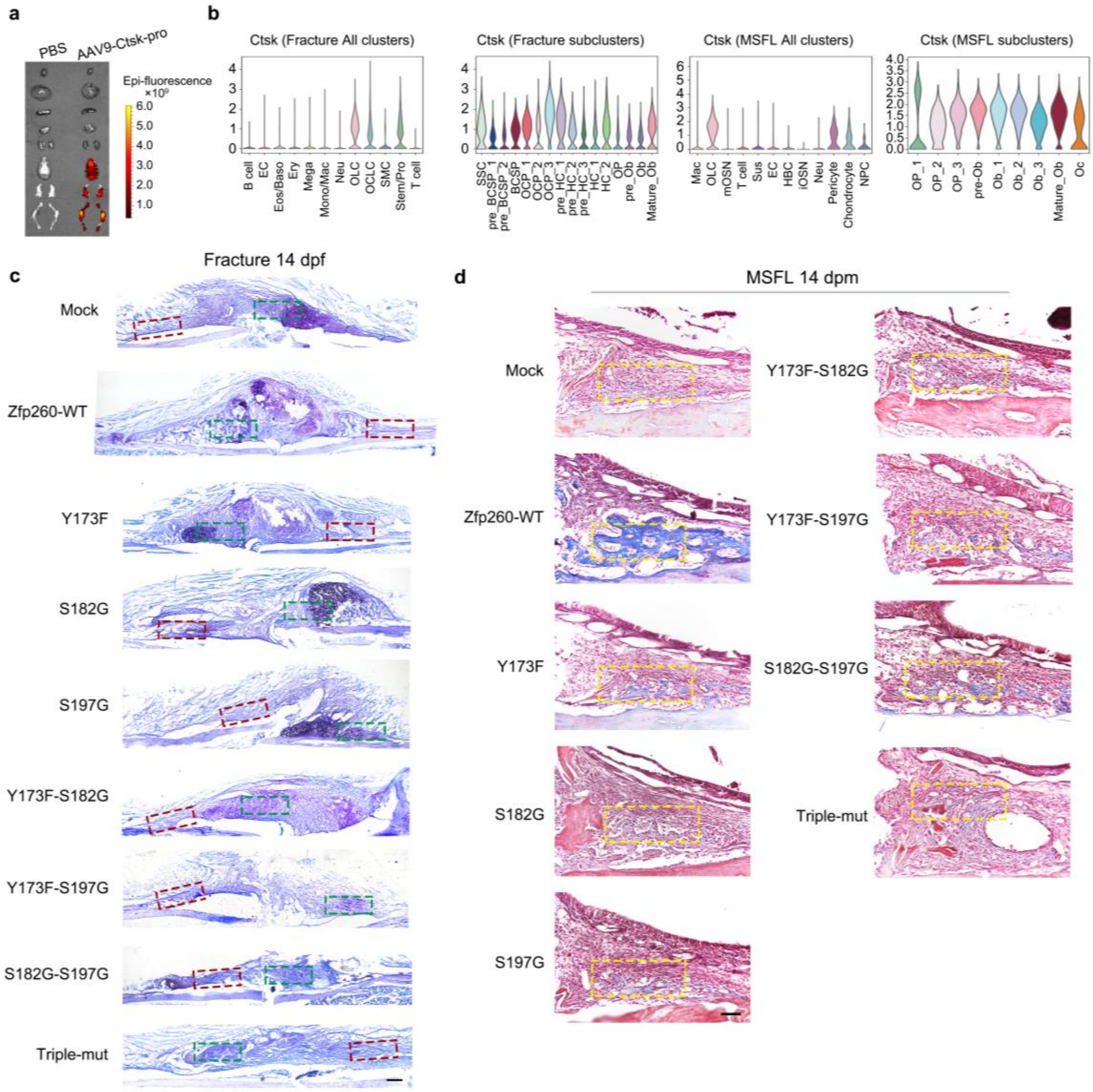

Supplementary Fig. 7 AAV9-Ctsk infection efficiency and low-magnification histology (corresponding to Figure 7)

a Distribution of immunofluorescent signals labeled with AAV9-*Ctsk*-ZsGreen, experiments were conducted 3 times independently, consistently producing similar results.

b Violin plots indicating the cellular distribution of *Ctsk* expression.

c Low-magnification Toluidine blue staining images of **Fig. 7g**, with red and blue dotted rectangles depicting the IO and EO regions, respectively. n = 4 from 4 biological replicates. Scalebars: 100 μm.

d Low-magnification Masson staining images of **Fig. 7h**, with the yellow dotted rectangles depicting the magnified region. n = 4 from 4 biological replicates. Scalebars: 50 μm.

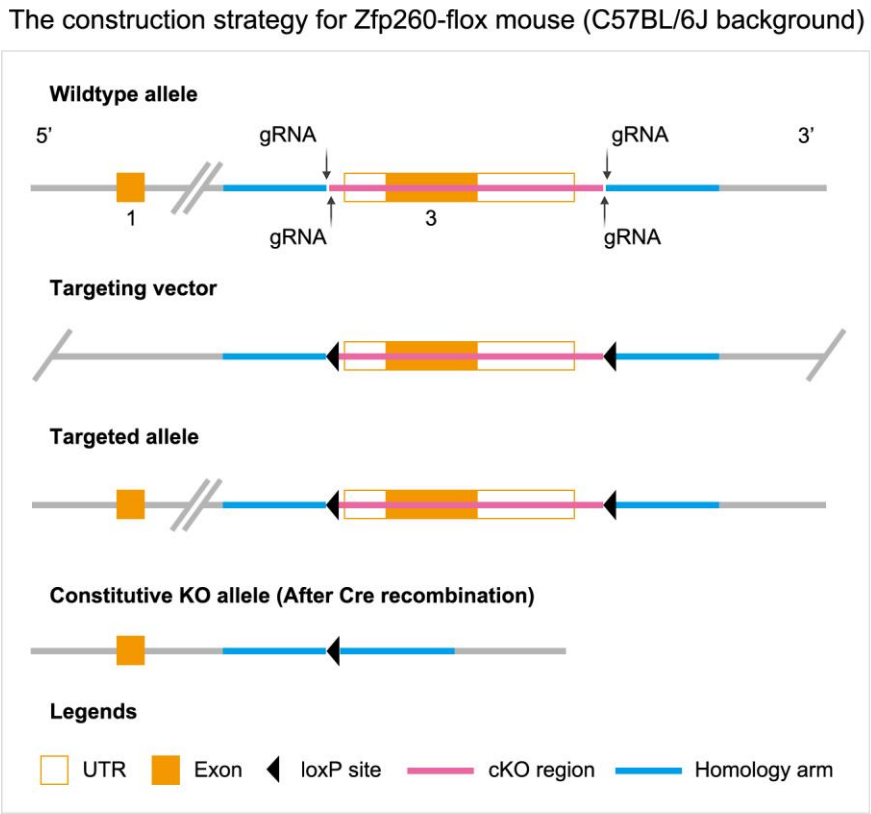

Supplementary Fig. 8 The construction strategy for *Zfp260-flox* mouse (C57BL/6J background)

The *Zfp260* gene (NCBI Reference Sequence: NM_011981; Ensembl: ENSMUSG00000049421) is located on mouse chromosome 7. Three exons have been identified, with the ATG start codon in exon 3 and the TAA stop codon in exon 3 (Transcript Zfp260-201: ENSMUST00000050735). Exon 3 will be designated as the conditional knockout region (cKO region). The region contains 1224 bp coding sequence. Deletion of this region should result in the loss of function of the mouse Zfp260 gene. To engineer the targeting vector, homologous arms and cKO region will be generated by PCR using BAC clone RP23-137H12 as the template. Ribonucleoprotein (RNP) and targeting vector will be co-injected into fertilized eggs for cKO mouse production.
