# Supplementary material for: Zfp260 choreographs the early stage osteo-lineage commitment of skeletal stem cells
Source: Nat Commun. 2024 Nov 24;15:10186. doi: 10.1038/s41467-024-54640-0 (PMC11586402; doi:10.1038/s41467-024-54640-0)
Supplement: Supplementary file 3 — Description of Additional Supplementary Files [file 41467_2024_54640_MOESM3_ESM.pdf]

Description of Additional Supplementary Files

Supplementary Data Legends:

Supplementary Data 1. List of primer sequences for qRT-PCR and ChIP-qPCR and the promoter sequence for constructing AAV9.

Supplementary Data 2. List of TFs screened from the scRNA-seq and bulk RNA-seq datasets of fracture and MSFL.

Supplementary Data 3. The comprehensive view of ATAC signals enriched Gene ontology terms. GO enrichment analysis is conducted using the hypergeometric test to assess the statistical significance of functional categories. Adjusted p-values (padj) are calculated using the Benjamini-Hochberg method to control for multiple comparisons.
