# Supplementary material for: Zfp260 choreographs the early stage osteo-lineage commitment of skeletal stem cells
Source: Nat Commun. 2024 Nov 24;15:10186. doi: 10.1038/s41467-024-54640-0 (PMC11586402; doi:10.1038/s41467-024-54640-0)
Supplement: Supplementary file 7 — Reporting Summary [file 41467_2024_54640_MOESM7_ESM.pdf]

nature portfolio

Corresponding author(s): Zuolin Wang

Last updated by author(s): Nov 1, 2024

Reporting Summary

Nature Portfolio wishes to improve the reproducibility of the work that we publish. This form provides structure for consistency and transparency in reporting. For further information on Nature Portfolio policies, see our Editorial Policies and the Editorial Policy Checklist.

Statistics

For all statistical analyses, confirm that the following items are present in the figure legend, table legend, main text, or Methods section.

n/a	Confirmed	
☐	☒	The exact sample size (*n*) for each experimental group/condition, given as a discrete number and unit of measurement
☐	☒	A statement on whether measurements were taken from distinct samples or whether the same sample was measured repeatedly
☐	☒	The statistical test(s) used AND whether they are one- or two-sided<br>*Only common tests should be described solely by name; describe more complex techniques in the Methods section.*
☒	☐	A description of all covariates tested
☐	☒	A description of any assumptions or corrections, such as tests of normality and adjustment for multiple comparisons
☐	☒	A full description of the statistical parameters including central tendency (e.g. means) or other basic estimates (e.g. regression coefficient) AND variation (e.g. standard deviation) or associated estimates of uncertainty (e.g. confidence intervals)
☐	☒	For null hypothesis testing, the test statistic (e.g. *F*, *t*, *r*) with confidence intervals, effect sizes, degrees of freedom and *P* value noted<br>*Give P values as exact values whenever suitable.*
☒	☐	For Bayesian analysis, information on the choice of priors and Markov chain Monte Carlo settings
☒	☐	For hierarchical and complex designs, identification of the appropriate level for tests and full reporting of outcomes
☒	☐	Estimates of effect sizes (e.g. Cohen's *d*, Pearson's *r*), indicating how they were calculated

Our web collection on statistics for biologists contains articles on many of the points above.

Software and code

Policy information about availability of computer code

Data collection	NIS-Elements software (v4.5000.1117.0) and LAS-X software (v4.7.0) were used to acquire confocal images. Real-time reverse transcription PCR was performed using QuantStudio Software by Thermo Scientific (v1.7.2). Western blots were detected using Amersham Imager 680 Analysis Software. RNA-seq, ChIP-seq and ATAC-seq were conducted on Illumina NovaSeq 6000 platform.
Data analysis	The micro-CT data was scanned by Micro-CT 50 (Scanco Medical), with its reconstruction and analysis by Scanco MicroCT Analysis Software. Statistical analyses were performed with SPSS 26.0 (IBM) software.<br>Single-cell RNA-sequencing analysis: The Cell Ranger toolkit v3.1 (10x Genomics) was applied to aggregate raw data, filter low-quality reads, align reads to the mouse reference genome (mm10), assign cell barcodes, and generate a unique molecular identifier (UMI) matrix. The raw UMI matrix was processed to exclude genes detected in fewer than 10 cells and cells with fewer than 200 genes. High-quality cells with thresholds of 500-120,000 UMIs and 400-8,000 genes were calculated. To exclude cells with a high mitochondrial proportion, we used the default parameter of 20%, ensuring that most of the heterogeneous cell types were included for downstream analysis. To minimize the effect of mitochondrial genes on subsequent analysis, a regression was performed prior to clustering. Scrublet was applied to remove potential doublets, with an expected doublet rate of 6%, and cells with doublet scores above 90% were excluded. Batch normalization was performed using CCA2, which models the UMI counts with a regularized negative binomial model to remove sequencing depth variation, while adjusting variance by pooling information across genes with similar abundances. Dimension reduction and unsupervised clustering were conducted following the workflow of the Python toolkit Scanpy. Briefly, dispersion-based methods were employed to detect the top 20 highly variable genes (HVGs). The normalized dispersion was obtained by scaling with the mean and standard deviation of the dispersions for genes. Percentages of mitochondrial gene counts and cell cycle-related gene counts were regressed out from the normalized expression matrices. For clustering, PCA was performed on the variable gene matrix to reduce noise. Fifty components were calculated, and the top 40 PCs were used for downstream analyses. Then, the Leiden algorithm was applied to identify cell clusters, with a resolution parameter of 1, referring to the construction of the TISCH database. Notably, the same principal components were also used for non-linear dimension reduction to

generate the Uniform Manifold Approximation and Projection (UMAP) for visualization.
Bulk RNA-sequencing analysis: The raw fastq files were trimmed and aligned using Hisat (https://www.psc.edu/resources/software/hisat-2) with the mm10 genome and nomarlized to TPM35. Mfuzz (http://mfuzz.sysbiolab.eu) was used for time course analysis. Cytoscape 3.7.1 (https://cytoscape.org/index.html) was used to depict the protein-protein interaction network. Principle component analysis (PCA) was applied to displayed the correlations among different cell types.
Analysis of ChIP-seq, CUT&Tag and ATAC-seq: The analysis was carried out using the HiChIP pipeline. FastQC and Cutadapt were used for quality control and adaptor trimming, respectively. Paired-end reads were mapped to the mm10 genome reference using Bowtie2, followed by duplicate removal with Sambamba. Narrow peaks were identified using the model-based analysis of ChIP-seq (MACS2) package with a cutoff of 1.00e-05. For data visualization, BEDTools and custom scripts were used to generate per-million read density profiles with a 200-bp window size and a 20-bp step size. Signal tracks were visualized using Integrative Genomics Viewer (IGV) software. The number of reads in the TSS±3kb region of all protein-coding genes was estimated and normalized to 10 million (RP10M), with log2 transformation and quantile normalization applied to each of the ChIP-seq, CUT&Tag, and ATAC-seq libraries. The read density (RPM, reads per milion) over the TSS±3kb region was calculated using the ngs.plot tool (v2.02). The binding motif was identified using MEME, and peak-annotated genes were used for the enrichment analysis of Gene Ontology-Biological Process (GO-BP) and the Kyoto Encyclopedia of Genes and Genomes (KEGG). The ROSE tool was used to identify super enhancers with the default settings, including the merging of remaining peaks within a distance of 12.5kb or less.

For manuscripts utilizing custom algorithms or software that are central to the research but not yet described in published literature, software must be made available to editors and reviewers. We strongly encourage code deposition in a community repository (e.g. GitHub). See the Nature Portfolio guidelines for submitting code & software for further information.

Data

Policy information about availability of data

All manuscripts must include a data availability statement. This statement should provide the following information, where applicable:

- Accession codes, unique identifiers, or web links for publicly available datasets
- A description of any restrictions on data availability
- For clinical datasets or third party data, please ensure that the statement adheres to our policy

All of the scRNA-seq, bulk RNA-seq, CUT&Tag, ChIP-seq and ATAC-seq data have been deposited in the Sequence Read Archive (SRA) under BioProject accession PRJCA026768 of the National Genomics Data Center (NGDC) database. The mass spectrometry data for GST and GST-Zfp260 pull-down assays reported in this study have been deposited in the NGDC OMIX database (OMIX ID: OMIX007435).

Research involving human participants, their data, or biological material

Policy information about studies with human participants or human data. See also policy information about sex, gender (identity/presentation), and sexual orientation and race, ethnicity and racism.

Reporting on sex and gender	N/A
Reporting on race, ethnicity, or other socially relevant groupings	N/A
Population characteristics	N/A
Recruitment	N/A
Ethics oversight	N/A

Note that full information on the approval of the study protocol must also be provided in the manuscript.

Field-specific reporting

Please select the one below that is the best fit for your research. If you are not sure, read the appropriate sections before making your selection.

☒ Life sciences ☐ Behavioural & social sciences ☐ Ecological, evolutionary & environmental sciences

For a reference copy of the document with all sections, see nature.com/documents/nr-reporting-summary-flat.pdf

Life sciences study design

All studies must disclose on these points even when the disclosure is negative.

Sample size	No statistical methods were used to predetermine the sample size. For micro-CT and histological analyses, n=4 (with AAV treatment) or n=6 mice of each groups (control vs cko) were analyzed and declared in corresponding figure legends. For FACS analysis of MSFL models, 15 mice per biological repeat, n=1 biological replicate was used. For FACS analysis of fracture models, 5 mice per biological repeat, n=2 biological replicates were carried out. This was shown to be sufficient in the previous studies, e.g. Deng, C. et al. Nat Commun 12, 2174 (2021); Sun, W. et al. Nat Commun 13, 2899 (2022); Jacome-Galazra, C.E. et al. Nature 568, 541-545 (2019); Weng, Y. et al. Cell Res 32, 814–830 (2022), to discern statistically significant differences. In molecular biology experiments, at least n=3 was chosen to generate p-values for the

determination of their significance, with exact sample size declared in corresponding figure legends.

Data exclusions	No data were excluded in this study.
Replication	The in vitro experiments reported in the manuscript were replicated independently at least three times to confirm reproducibility. The exact sample size in terms of mice in all experiments are declared in corresponding figure legends.
Randomization	Samples and mice were randomly allocated to different groups.
Blinding	Analyses were done objectively. Blinding was not relevant for this study.

Reporting for specific materials, systems and methods

We require information from authors about some types of materials, experimental systems and methods used in many studies. Here, indicate whether each material, system or method listed is relevant to your study. If you are not sure if a list item applies to your research, read the appropriate section before selecting a response.

Materials & experimental systems

n/a	Involved in the study	
☐	☒	Antibodies
☐	☒	Eukaryotic cell lines
☒	☐	Palaeontology and archaeology
☐	☒	Animals and other organisms
☒	☐	Clinical data
☒	☐	Dual use research of concern
☒	☐	Plants

Methods

n/a	Involved in the study	
☐	☒	ChIP-seq
☐	☒	Flow cytometry
☒	☐	MRI-based neuroimaging

Antibodies

Antibodies used

Antibodies used in mIHC (dilution 1:400 for all antibodies) included goat anti-mouse/human/rat Itgav (AF1219, Novus Biologicals), mouse anti-mouse/rat CD90 (NB100-65543, Novus Biologicals), mouse anti-mouse/human CD105 (NBP2-22122, Novus Biologicals), rabbit anti-human/mouse/rat CD200 (AF2724, Novus Biologicals), rabbit anti-mouse/human/rat Runx2 (ab236639, Abcam), rabbit anti-mouse/human/rat Sox9 (ab185966, Abcam), rabbit anti-mouse/human Alpl (MA5-24845, Invitrogen), rabbit anti-mouse/human/rat Zfp260 (ABE295, Merck), mouse anti-human/mouse/rat p300 (NB100-616, Novus Biologicals), rabbit anti-human/mouse MED1 (NB100-2574, Novus Biologicals), rabbit anti-human/mouse BRD4 (NBP2-76393, Novus Biologicals), mouse anti-human/mouse/rat Prkca (NB600-201, Novus Biologicals), rabbit anti-V5 tag (13202, CST), mouse anti-Collagen type I (67288-1-Ig, proteintech), rabbit anti-Collagen type II (28459-1-AP, proteintech). The tyramide reagents used in mIHC included AF350 (B40952, Invitrogen), AF488 (B40953, Invitrogen), AF546 (B40954, Invitrogen), AF594 (B40957, Invitrogen) and AF647 (B40958, Invitrogen).

Antibodies used in WB at a 1:1000 dilution included: mouse anti-p300 (NB100-616, Novus Biologicals), rabbit anti-MED1 (NB100-2574, Novus Biologicals), rabbit anti-BRD4 (NBP2-76393, Novus Biologicals), rabbit anti-V5 tag (13202, CST), rabbit anti-Flag tag (14793, CST), mouse anti-Prkca (NB600-201, Novus Biologicals), rabbit anti-β Actin (4970, CST), rabbit anti-Histone H3 (4499, CST). The VeriBlot for IP Detection reagent (conjugated with HRP, ab131366, Abcam) was used at a 1:200 dilution for the detection of immunoprecipitated samples. For other samples, conventional HRP-conjugated secondary antibodies specific to the corresponding species were diluted 1:2000 for WB signal detection.

V5 tag antibody (13202, CST, 1:100), Flag tag antibody(14793, CST, 1:100), or normal Rabbit IgG (2729, CST, the same concentration as the specific target antibody) were used for immunoprecipitation. Immune complexes were subjected to immunoblotting with V5 tag antibody (13202, CST, 1:1000), Flag tag antibody(14793, CST, 1:1000), BRD4 antibody (NBP2-76393, Novus Biologicals, 1:1000), p300 antibody (NB100-616, Novus Biologicals, 1:1000), MED1 antibody (NB100-2574, Novus Biologicals, 1:1000), Prkca antibody (NB600-201, Novus Biologicals, 1:1000).

H3K27ac antibody (8173, CST, 1:100) or H3K4me1 (5326, CST, 1:100) were used for CUT & Tag. A guinea pig anti-rabbit secondary antibody (provided in the kit 53160, Active Motif) was diluted in 1:100 and used for incubation.

V5 tag antibody (ab15828, Abcam, 1:100), H3K27ac antibody (8173, CST, 1:100), Brd4 antibody (13440, CST, 1:100) with normal Rabbit IgG (2729, CST, the same concentration as the specific target antibody) as a non-specific IgG control were used for chromatin immunoprecipitation.

Validation

All antibodies were commercial available and characterized by manufacturers online.
1. V5 tag antibody (https://www.abcam.cn/products/primary-antibodies/v5-tag-antibody-ab15828.html)
2. V5 tag antibody (https://www.cellsignal.cn/products/primary-antibodies/v5-tag-d3h8q-rabbit-mab/13202)
3. H3K4me1 antibody (https://www.cellsignal.cn/products/primary-antibodies/mono-methyl-histone-h3-lys4-d1a9-xp-rabbit-mab/5326)
4. H3K27ac antibody (https://www.cellsignal.cn/products/primary-antibodies/acetyl-histone-h3-lys27-d5e4-xp-rabbit-mab/8173)
5. Brd4 antibody (https://www.cellsignal.cn/products/primary-antibodies/brd4-e2a7x-rabbit-mab/13440)
6. Brd4 antibody (https://www.novusbio.com/products/brd4-antibody-bl-149-2h5_nbp2-76393)

7. Flag tag antibody (https://www.cellsignal.cn/products/primary-antibodies/dykddddk-tag-d6w5b-rabbit-mab-binds-to-same-epitope-as-sigma-aldrich-anti-flag-m2-antibody/14793)
8. p300 antibody (https://www.novusbio.com/products/kat3b-p300-antibody-rw105_nb100-616)
9. MED1 antibody (https://www.novusbio.com/products/trap220-med1-antibody_nb100-2574)
10. Prkca antibody (https://www.novusbio.com/products/pkc-alpha-antibody-mc5_nb600-201)
11. β-Actin antibody (https://www.cellsignal.cn/products/primary-antibodies/b-actin-13e5-rabbit-mab/4970)
12. Histone H3 antibody (https://www.cellsignal.cn/products/primary-antibodies/histone-h3-d1h2-xp-rabbit-mab/4499)
13. Itgav antibody (https://www.novusbio.com/products/integrin-alpha-v-cd51-antibody_af1219)
14. CD90 antibody (https://www.novusbio.com/products/cd90-thy1-antibody-ox-7_nb100-65543)
15. CD105 antibody (https://www.novusbio.com/products/endoglin-cd105-antibody-3a9_nbp2-22122)
16. CD200 antibody (https://www.novusbio.com/products/cd200-ox2-antibody_af2724)
17. Runx2 antibody (https://www.abcam.cn/products/primary-antibodies/runx2-antibody-epr22858-106-chip-grade-ab236639.html)
18. Sox9 antibody (https://www.abcam.cn/products/primary-antibodies/sox9-antibody-epr14335-78-ab185966.html)
19. Alpl antibody (https://www.thermofisher.cn/cn/zh/antibody/product/ALPL-Antibody-clone-3G2-Recombinant-Monoclonal/MA5-24845)
20. Zfp260 antibody (https://www.emdmillipore.com/US/en/product/Anti-Zinc-Finger-Protein-260-Antibody,MM_NF-ABE295?ReferrerURL=https%3A%2F%2Fcn.bing.com%2F&bd=1)
21. Collagen type I antibody (https://www.ptgcn.com/products/Collagen-I-Antibody-67288-1-Ig.htm)
22. Collagen type II antibody (https://www.ptgcn.com/products/Collagen-Type-II-Antibody-28459-1-AP.htm)
23. Rabbit IgG Horseradish Peroxidase-conjugated antibody (https://www.rndsystems.com/cn/products/rabbit-igg-horseradish-peroxidase-conjugated-antibody_haf008)
24. Mouse IgG Horseradish Peroxidase-conjugated Antibody (https://www.rndsystems.com/cn/products/mouse-igg-horseradish-peroxidase-conjugated-antibody_haf007)
25. VeriBlot for IP Detection (https://www.abcam.cn/products/reagents/veriblot-for-ip-detection-reagent-hrp-ab131366.html)
26. Normal Rabbit IgG (https://www.cellsignal.cn/products/primary-antibodies/normal-rabbit-igg/2729)
27. Tyramide reagent AF350 (https://www.thermofisher.cn/order/catalog/product/B40952)
28. Tyramide reagent AF488 (https://www.thermofisher.cn/order/catalog/product/B40953)
29. Tyramide reagent AF546 (https://www.thermofisher.cn/order/catalog/product/B40954)
30. Tyramide reagent AF594 (https://www.thermofisher.cn/order/catalog/product/B40957)
31. Tyramide reagent AF647 (https://www.thermofisher.cn/order/catalog/product/B40958)

Eukaryotic cell lines

Policy information about cell lines and Sex and Gender in Research

Cell line source(s)	293T cell line was ordered from the cell bank of Cyagen Biosciences (OriCell®293T, H4-1401).
Authentication	The cell line has been validated using the short tandem repeat (STR) profiling method.
Mycoplasma contamination	The cell line has been tested negative for mycoplasma contamination.
Commonly misidentified lines (See ICLAC register)	No commonly misidentified cell lines were used.

Animals and other research organisms

Policy information about studies involving animals; ARRIVE guidelines recommended for reporting animal research, and Sex and Gender in Research

Laboratory animals	Igs2em1(CAG-LSL-ZsGreen-wpre-pA-CAG-RSR-tdTomato-wpre-pA)Smoc (H11LSL-ZsGreen-RSR-tdTomato) (NM-KI-200319), Ctskem1(2A-CreERT2-WPRE-polyA)Smoc (CtskcreER) (NM-KI-200067) with C57BL/6J background were purchased from the Shanghai Model Organisms Center, China. C57BL/6J-Zfp260em1Cflox/Cya (Zfp260flox/flox) (S-CKO-09613) was obtained from Cyagen, USA. All mice were used for analysis regardless of sex. Animals were housed at the Laboratory Animal Center of Tongji University with constant temperature and humidity in a 12-h light cycle. For descriptive and functional studies, 7wk-old mice were applied, with the exact ages of animals used in each experiment are listed in the figure legend accordingly.
Wild animals	Our study did not involve wild animals.
Reporting on sex	All mice were used for analysis regardless of sex.
Field-collected samples	Our study did not involve samples collected from the filed.
Ethics oversight	All mouse procedures were approved by the Institutional Animal Care and Use Committee of Tongji University.

Note that full information on the approval of the study protocol must also be provided in the manuscript.

Plants

Seed stocks: N/A

Novel plant genotypes: N/A

Authentication: N/A

ChIP-seq

Data deposition

☒ Confirm that both raw and final processed data have been deposited in a public database such as GEO.

☒ Confirm that you have deposited or provided access to graph files (e.g. BED files) for the called peaks.

Data access links
May remain private before publication.

https://ngdc.cncb.ac.cn/gsa/browse/CRA019163;
https://ngdc.cncb.ac.cn/bioproject/browse/PRJCA026768

Files in database submission

CRR1302892 ChIP-BF-ctrl-input
CRR1302893 ChIP-BF-cko-input
CRR1302894 ChIP-MSFL-ctrl-input
CRR1302895 ChIP-MSFL-cko-input
CRR1302896 K4-BF-ctrl1
CRR1302897 K4-BF-ctrl2
CRR1302898 K4-BF-ctrl3
CRR1302899 K4-BF-cko1
CRR1302900 K4-BF-cko2
CRR1302901 K4-BF-cko3
CRR1302902 K4-MSFL-ctrl1
CRR1302903 K4-MSFL-ctrl2
CRR1302904 K4-MSFL-ctrl3
CRR1302905 K4-MSFL-cko1
CRR1302906 K4-MSFL-cko2
CRR1302907 K4-MSFL-cko3
CRR1302908 K27-BF-ctrl1
CRR1302909 K27-BF-ctrl2
CRR1302910 K27-BF-ctrl3
CRR1302911 K27-BF-cko1
CRR1302912 K27-BF-cko2
CRR1302913 K27-BF-cko3
CRR1302914 K27-MSFL-ctrl1
CRR1302915 K27-MSFL-ctrl2
CRR1302916 K27-MSFL-ctrl3
CRR1302917 K27-MSFL-cko1
CRR1302918 K27-MSFL-cko2
CRR1302919 K27-MSFL-cko3
CRR1302920 ChIP-PSC-input
CRR1302921 ChIP-PSC-V5-1
CRR1302922 ChIP-PSC-V5-2

Genome browser session (e.g. UCSC): No longer applicable.

Methodology

Replicates: CUT&Tag experiments were performed in triplicate per condition per experiment. ChIP-seq experiments were performed in duplicate per condition per experiment.

Sequencing depth: Each CUT&Tag and ChIP-seq with pooled more than 10 M uniq-mapped reads.

Antibodies: H3K27ac antibody (8173, CST, 1:100), H3K4me1 (5326, CST, 1:100) , V5 tag antibody (ab15828, Abcam, 1:100), Brd4 antibody (13440, CST, 1:100)

Peak calling parameters: Peaks were called by MACS2 with the parameters --nolambda --nomodel.

Data quality	CRR1302896 - CRR1302898 K4-BF-ctrl-reps1-3 with average called peaks > 50,000 by MACS2, q-value cutoff 0.05<br>CRR1302899 - CRR1302901 K4-BF-cko-reps1-3 with average called peaks > 50,000 by MACS2, q-value cutoff 0.05<br>CRR1302902 - CRR1302904 K4-MSFL-ctrl-reps-1-3 with average called peaks > 50,000 by MACS2, q-value cutoff 0.05<br>CRR1302905 - CRR1302907 K4-MSFL-cko-reps-1-3 with average called peaks > 50,000 by MACS2, q-value cutoff 0.05<br>CRR1302908 - CRR1302910 K27-BF-ctrl-reps-1-3 with average called peaks > 12,000 by MACS2, q-value cutoff 0.05<br>CRR1302911 - CRR1302913 K27-BF-cko-reps-1-3 with average called peaks > 12,000 by MACS2, q-value cutoff 0.05<br>CRR1302914 - CRR1302916 K27-MSFL-ctrl-reps-1-3 with average called peaks > 12,000 by MACS2, q-value cutoff 0.05<br>CRR1302917 - CRR1302919 K27-MSFL-cko-reps-1-3 with average called peaks > 12,000 by MACS2, q-value cutoff 0.05<br>CRR1302921 ChIP-PSC-V5-1 called peaks 4432 by MACS14, p-value cutoff 1.00e-05<br>CRR1302922 ChIP-PSC-V5-2 called peaks 6777 by MACS14, p-value cutoff 1.00e-05
Software	FastQC v0.11.8, cutadapt v4.5, Bowtie 2 v2.4.4, sambamba v0.6.6, macs2 v2.2.9.1, macs14 v1.4.2

Flow Cytometry

Plots

Confirm that:

☒ The axis labels state the marker and fluorochrome used (e.g. CD4-FITC).

☒ The axis scales are clearly visible. Include numbers along axes only for bottom left plot of group (a 'group' is an analysis of identical markers).

☒ All plots are contour plots with outliers or pseudocolor plots.

☒ A numerical value for number of cells or percentage (with statistics) is provided.

Methodology

Sample preparation	For the analysis of SSC lineage cells, the callus of fracture mice (5 mice per biological repeat, n=2 per time point), along with the newly formed tissues of MSFL models (15 mice per biological repeat, n=1 per time point) were dissected under stereomicroscopy.<br><br>Tissues were digested in DPBS containing 0.3% collagenase, 0.3% dispase II and 0.01% DNase I at 37 °C for 30 min in a shaking bath, followed by filtering with 70 μm cell strainers (352350, BD Falcon). After centrifugation at 4 °C at 300g for 10 min, the supernatant was discarded, and cells were suspended in red blood cell lysis buffer (00433357, eBioscience) at 4 °C for 5 min. After neutralization and centrifugation, the cell pellets were resuspended by staining buffer containing the CD16/32 blocking antibody (156603, Biolegend) and incubated for 30 min, followed by staining with antibodies. Cells were incubated in the dark for 1 hour on ice with primary antibody solution, washed 2 to 3 times with FACS buffer, and incubated with secondary antibody solution for 20 minutes.<br><br>All antibodies were diluted at 1:200 except for the anti-CD45 antibody, which was diluted at 1:800. Antibodies used included: CD140a (Pdgfra)-BV605 (APA5, Biolegend), CD31-PE/DazzleTM 594 (MEC13.3, Biolegend), CD45-PE/DazzleTM 594 (30-F11, Biolegend), TER119-PE/DazzleTM 594 (TER-119, Biolegend), CD105-APC/Cy7 (MJ7/18, Biolegend), CD200-APC (OX-90, Biolegend), CD51 (Itgav)-biotin (RMV-7, Biolegend), BV421-Streptavidin (405226, Biolegend), Ly-51-PE/Cy7 (6C3, Biolegend), CD90.1 (Thy1.1)-PerCP/Cy5.5 (OX-7, Biolegend), CD90.2 (Thy1.2)-PerCP/Cy5.5 (30-H12, Biolegend). Cells were then washed several times and re-suspended in FACS buffer, followed by staining with Zombie Aqua, to distinguish live and dead cells with incubation for 20 minutes.
Instrument	BD FACSAriaTM III was applied for cell sorting, while the BD-LSRFortessa was used for FACS analysis.
Software	FlowJo (version 10.8.1)
Cell population abundance	Sorting was performed using a 4-way collection, and was validated to result in >95% purity of the intended population in postsort fractions.
Gating strategy	Fluorescence minus one (FMO) controls were used for additional compensation and to assess background levels for each stain. Gates were drawn as determined by internal FMO controls to separate positive and negative populations for each cell surface marker. We selected live cells (neagtive for Zombie Aqua) and excluded doublets. Mesenchymal cell populations negative for CD45, CD31 and Ter119 cell surface markers were then analyzed according to the approach described in Supplementary figure 4.

☒ Tick this box to confirm that a figure exemplifying the gating strategy is provided in the Supplementary Information.
